# Coronavirus Disease 2019 (COVID-19) in Children: Vulnerable or Spared? A Systematic Review

**DOI:** 10.7759/cureus.8207

**Published:** 2020-05-20

**Authors:** Hajra Saleem, Jawaria Rahman, Nida Aslam, Salikh Murtazaliev, Safeera Khan

**Affiliations:** 1 Family Medicine, California Institute of Behavioral Neurosciences and Psychology, Fairfield, USA; 2 Internal Medicine, California Institute of Behavioral Neurosciences and Psychology, Fairfield, USA; 3 Dermatology, California Institute of Behavioral Neurosciences and Psychology, Fairfield, USA

**Keywords:** corona virus, covid-19 in children, covid-19 outbreak, covid-19, novel corona virus, covid-19 in neonates, sars-cov-2 in pediatric patients, sars-cov-2 (severe acute respiratory syndrome coronavirus -2)

## Abstract

The ongoing pandemic of coronavirus disease 2019 (COVID-19) has affected people from all cultures, religions, gender, and age groups around the world. In the last few months, several studies have been conducted on various aspects of COVID-19. Our goal was to see if the pediatric population is vulnerable to this infection.

In this review, we conducted extensive research mainly by using the PubMed database. We used Medical Subject Headings (MeSH) and associated keywords to engage in an extensive search focussing on COVID-19 in the pediatric population. We discovered that most of the studies were from China, and some of them were in the Chinese language. However, English translations of many of the studies were available. For accessing the relevant statistical data, we relied on the World Health Organization (WHO) resources and the official website of the Ontario Government (ontario.ca).

Most of the studies showed that the virus has affected the pediatric population. However, we found some differences among these studies regarding the severity of symptoms in children affected by COVID-19. While a few studies stated that the virus has presented with milder symptoms in the pediatric population, some studies have presented data of children who have suffered life-threatening complications due to COVID-19.

Although the data is limited, we have been able to conclude from the studies we reviewed that COVID-19 does indeed affect children the same way as any other age group. Moreover, children can act as carriers of the virus and can endanger the lives of other individuals. Besides, neonates and infants can easily acquire the infection from family members without having any exposure to the outside world. Hence, utmost care should be taken while handling this population. More trials and studies should be conducted to analyze the impact of early diagnosis of infection in children and its management.

## Introduction and background

Coronavirus disease 2019 (COVID-19), a respiratory infection that originated in Wuhan, China, is the talk of the town today [1]. It is caused by a virus that belongs to a family of viruses called coronaviruses and has been responsible for 169,006 deaths so far [2]. Coronaviruses are of different types [e.g., severe acute respiratory syndrome coronavirus (SARS-CoV), Middle East respiratory syndrome coronavirus (MERS-CoV)], and have caused serious human illnesses [3]. SARS-CoV-2, the virus that causes COVID-19, is a newer form, which has been newly diagnosed in humans. It is an enveloped RNA virus with positive-sense RNA genomes ranging from 25.5 to 32 KB in length. It is spherical in shape and ranges from 70-120 nm in diameter with four structural proteins. The viral envelope is covered by characteristic spike-shaped glycoproteins (S) as well as the envelope (E) and membrane (M) proteins [4]. Studies have suggested that it can spread through close contacts and nosocomial secretions by coughing or sneezing. Furthermore, the fecal-oral route has also been suggested as a mode of transmission [5]. People can protect themselves from being infected by washing hands frequently, avoiding touching the face, and avoiding close contacts, especially with people who are sick.

COVID-19 has mostly caused mild to moderate respiratory illnesses in humans, and infected individuals usually recover without requiring any special treatment. The population that is at the highest risk is elderly people, especially those with existing ailments like diabetes, cardiac problems, underlying respiratory problems, cancers, or any other immunosuppressed issues [6]. Hence, increased mortality has been seen in the older age group. COVID-19 has affected children too. Recent data have suggested that children are more likely to have milder symptoms. Among the children who were tested positive, 45% showed typical symptoms, and 42% presented with mild respiratory symptoms. While 13% were asymptomatic, no child presented with life-threatening symptoms [7]. Children with underlying medical conditions like asthma or diabetes may be at higher risk of more severe disease; infants can have a higher risk too. In general, children do not seem to be at high risk, but they can spread the virus, which should be prevented by taking adequate precautions, such as avoiding playdates, along with other precautions mentioned earlier [8]. There is very limited data pertaining to children who are infected with COVID-19, including infected neonates and infants. In this review, we summarize all the data that has been collected so far and engage in a systematic review of the same.

While most of the people/studies think/suggest that children are usually spared by the coronavirus and present only with milder symptoms even if infected, there are studies that prove that COVID-19 has affected children with the same severity as any other patient population. For instance, a 55 day-old female infant in China was reported to be positive, requiring hospital admission [9]. She was severely sick with decreased arterial oxygen partial pressures and elevated lactic acid. Another case of a one-year-old boy was reported from Wuhan’s Children's Hospital. He was the first critically ill pediatric patient to be diagnosed with COVID-19, and he had presented with diarrhea, vomiting, and shortness of breath [10]. There is no confirmed data yet regarding the vertical transmission of COVID-19. However, perinatal COVID-19 infections may lead to fetal distress, premature labor, respiratory distress, thrombocytopenia accompanied by abnormal liver function, and even death [11].

The typical radiographic findings from chest CT scans were similar to those of adults but were milder. Patchy ground-glass opacities and consolidations were seen because of the parenchymal destruction in children with proven COVID-19, which all normalized during treatment [12]. There is no particular treatment or vaccine so far, and the use of antivirals is still being debated. It has been reported that the following five drugs can be used by weighing the benefits and drawbacks: interferon-alpha, lopinavir/ritonavir, ribavirin, chloroquine diphosphate, and umifenovir [13]. High-dose pulmonary surfactant, nitric oxide inhalation, and high-frequency oscillatory ventilation are the other treatment options for newborns. The role of antibiotics is limited to proven bacterial infections only [14]. The goal of this review article was to delve deeper and find out if any child, irrespective of age, suffered from any severe complications or death due to COVID-19. Moreover, we also wanted to create awareness that children are equally susceptible to this infection as any other individual. Consequently, children could be saved from getting this infection and can be stopped from spreading the infection as silent carriers.

## Review

Methods

We conducted this systematic review by using Preferred Reporting Items for Systematic Reviews and Meta-Analyses (PRISMA) guidelines. For data search, we used different search engines; we predominantly used PubMed for collecting most of the data. Resources such as Google Scholar, MEDLINE, PubMed Central, the WHO website, the official website of the Government of Canada (canada.ca), WebMD, and some grey literature were also used. We carried out our research by using the following Medical Subject Headings (Mesh) terms and keywords: “COVID-19,” “children,” “acute respiratory distress syndrome,” “coronavirus in children,” and “novel coronavirus.” We selected research papers from last year with children as the target population. However, a few articles showing a comparison between different age groups were also included. We focused on children from all around the world without any gender discrimination. A quality check was also done for all the selected studies by using the assessment of multiple systematic reviews (AMSTAR) checklist, and four articles were removed. All the remaining studies and reviewed articles collected were found to be establishing the relationship between COVID-19 and the pediatric population.

The inclusion and exclusion criteria were applied. Out of the 42 studies conducted in the past year, 34 were selected; articles about coronavirus in the general population were excluded. The selected studies were all peer-reviewed. We used the English translation for two articles that were originally in Chinese. We used the full-text version of the articles, and review was done scientifically and within the ethical boundaries.

Results

Most of the research was carried out by using PubMed and Google scholar. Various keywords such as “COVID-19,” “novel coronavirus in children,” “COVID-19 in children,” and “acute respiratory distress syndrome” were used. Due to the recency of the topic, the search engines returned very limited data; using Mesh keywords COVID-19 in children yielded three articles, while rest were collected through keywords only.

Out of the 42 articles, eight were removed when we applied the inclusion/exclusion criteria (full text, peer-reviewed, one-year duration, pediatric population). A total of 34 studies that fulfilled the criteria of COVID-19 in children were finalized after the quality check. For accessing the relevant statistical data, we relied on the World Health Organization (WHO) resources and the official website of the Ontario Government (ontario.ca). All studies were in English, except for two that were in Chinese. Since English translation for these two was available, we included them in our review. We did not set any restrictions regarding the country of origin of articles/cases. However, the majority of the data was from China, Korea, and Iran.

Almost all the studies documented the occurrence of COVID-19 in children. While some articles stated that the disease is milder in the pediatric population, there were other studies that documented fatal cases in children (Table 1).

**Table 1:** Selected studies in the review COVID-19: coronavirus disease 2019

First author	Year	Study purpose	Result/conclusion
Cui Y [9]	2020	COVID-19-positive infant with complications	Pediatric population can also present with life-threatening complications
Hong H [14]	2020	Coronavirus infection symptoms in newborns, infants, and children	Children are susceptible to COVID-19 infection
Lee PI [27]	2020	To see whether children are prone to COVID-19	Out of 9 infected patients, 4 had a fever, 2 had a mild upper respiratory infection, 1 no symptoms but tested positive, and there was no information on symptoms for two
Kam KQ [30]	2020	High viral load in a healthy infant	It was found out that a 6-month old infant had a persistently positive test for COVID-19 without having any symptoms, putting him at high risk for spreading infection
Ji LN [24]	2020	Clinical features of COVID-19 in children	Children infected with COVID-19 were having milder symptoms than the adults who were infected
Wang Y [13]	2020	Role of antivirals in COVID-19 children	Antivirals should be given cautiously in children after weighing the benefits and drawbacks

Discussion

Virology and Pathogenesis/Biology and Virology

Coronavirus belongs to the family Coronaviridae, which has two sub-types: Coronavirinae and Torovirinae. These are enveloped RNA viruses with positive-sense genomes and covered by spike-shaped glycoproteins. These spike proteins help the virus to enter the host cells [4]. According to one of the studies done by Hong et al., this human coronavirus is also known as SARS-CoV-2 and COVID-19, the latter name given by WHO is a rapidly emerging beta coronavirus and is extremely infectious [14]. The virus invades multiple respiratory epithelial cell types, alveolar macrophages, and monocytes by using the host ACE2 receptors [15,16]. The median incubation period of the virus is estimated to be three days (range: 0-24 days) [17].

Mode of Transmission

**Figure 1:**
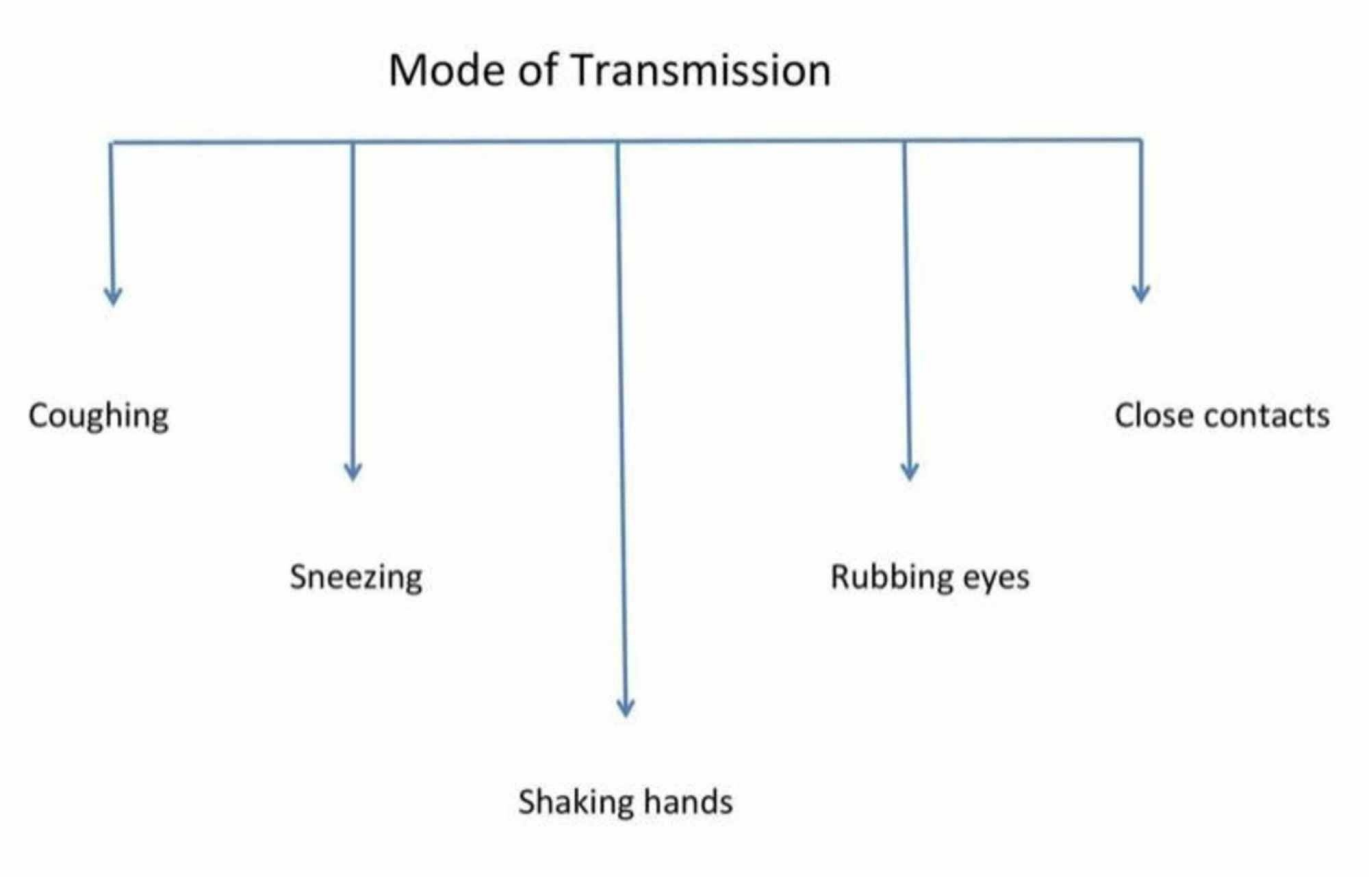
COVID-19 - modes of transmission COVID-19: coronavirus disease 2019

According to WHO, coronavirus is known to spread through droplets from nasal secretions, especially when a COVID-19 patient coughs or sneezes, or through saliva [6]. Also, there is a chance of getting the infection if an infected person speaks loudly and moistly or when you rub the eyes with contaminated hands [18]. Another study has stated that the infection spreads through close contacts and respiratory droplets [14]. Until now, no transplacental transmission of infection from mother to infant has been reported [19]. Our review has also shown that the fecal-oral route is also responsible for the transmission of the novel coronavirus, which was detected in the stool samples of the very first case in the United States [20]. The various modes of transmission are presented in Figure 1.

Clinical Course

The novel coronavirus-associated pneumonia, which is now known as COVID-19, can present with various symptoms ranging from mild to moderate to severe. Besides common symptoms as mentioned above, patients may also develop a rapid heartbeat, shortness of breath, chest pain, dizziness, or increased sweating. Those who have pneumonia can also develop acute respiratory distress syndrome [21]. Clinical presentation can vary from patient to patient depending on the immunity status. Individuals with underlying medical conditions like diabetes and asthma show more severe symptoms as compared to healthy individuals [8]. Some patients may also develop gastrointestinal symptoms like nausea, vomiting, abdominal pain and discomfort, and diarrhea [14].

**Figure 2:**
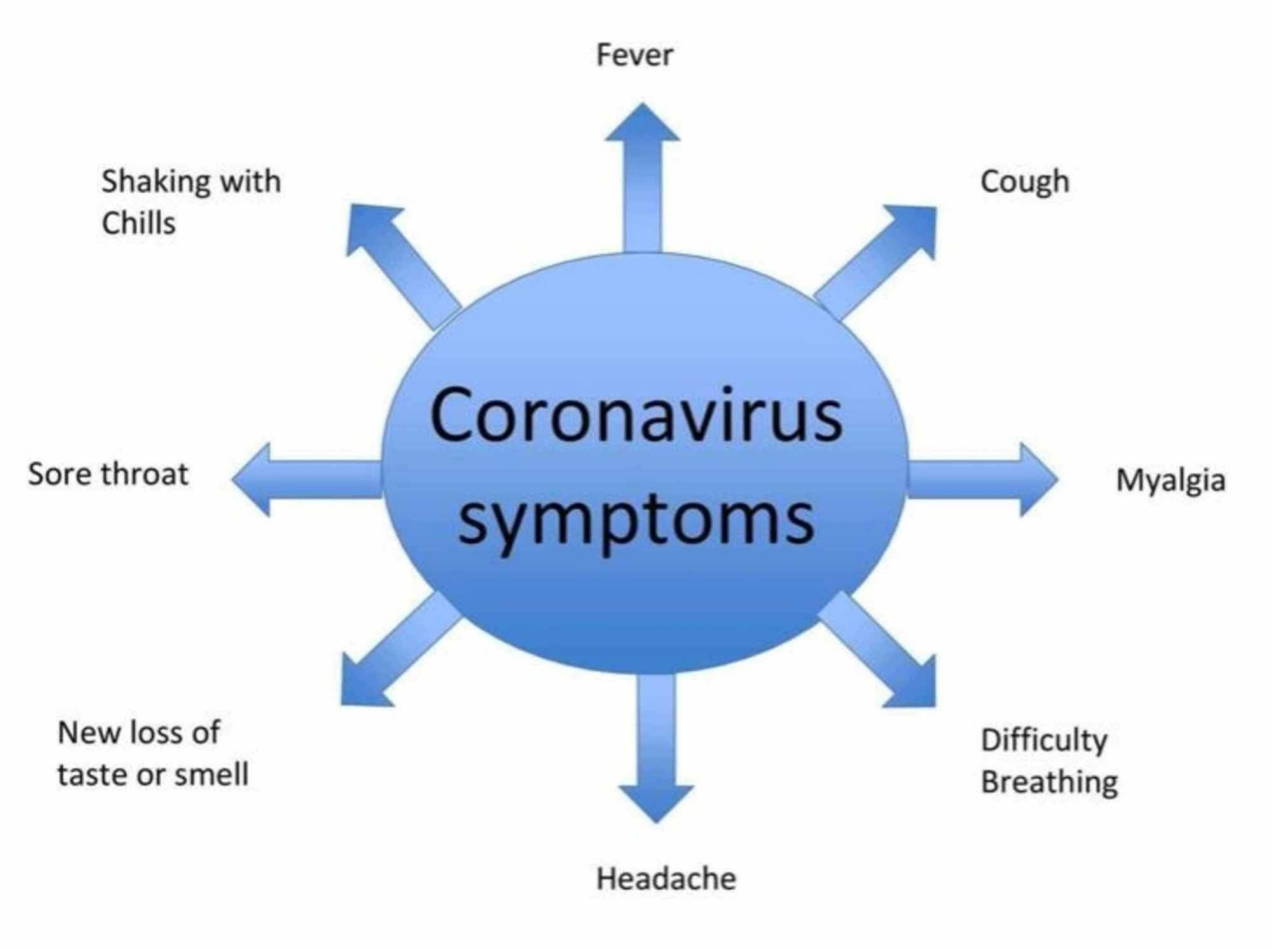
Common symptoms of COVID-19 COVID-19: coronavirus disease 2019

According to one study, in severely ill pediatric patients, the most common symptoms in descending order were as follows: polypnea, fever, cough, expectoration, nausea/vomiting, diarrhea, fatigue/myalgia, headache, and constipation [22]. Some patients can also deteriorate further, and in such cases, dyspnea and cyanosis can occur along with irritability, restlessness, septic shock, metabolic acidosis, or coagulation disorder (Figure 2) [18].

Susceptibility of Children to COVID-19

It is now proven that Coronavirus does not spare any age group. It has affected children, adults, and the elderly in almost similar patterns. To be more specific, neonates, infants, and older children of all ages are affected. The severity of symptoms usually depends on the immunity of the individual child and previous health status of the child, but it is not true in all cases. However, limited data is available at this point to reach a definite conclusion regarding various aspects of COVID-19 infection in children.

A study done in China documented the first case of a severely ill one-year-old boy with COVID-19 pneumonia. He had been previously healthy and up to date with immunizations. He presented with unique symptoms of intermittent diarrhea, vomiting, and fever with shortness of breath and later on developed acute respiratory distress syndrome and septic shock accompanied by acute renal failure [10]. Another study discussed a 55-day old baby who got infected with COVID-19 and presented with multiple organ failure [9]. On the other hand, data has also suggested that approximately 90% of children who were infected had mild to moderate symptoms, and only a few required hospitalization [8].

In addition to the severity of symptoms, studies were also done to see the sources of infection in children. In one study, data were collected for nine infants who got infected and were admitted to hospitals in China. It was revealed that infected family members living together could be a source for transmission, as these small babies cannot wear masks. Luckily, none of them required mechanical ventilation [23]. Similarly, there were two more cases of infected children, who were in close family contact and presented with milder symptoms [24]. One of the Iranian studies demonstrated infected pediatric cases with a history of one infected family member [25]. In Korea, the very first pediatric case involved a 10-year old female child with milder symptoms and, again, a history of close contact with an infected family member [26].

Some studies have also suggested that most of the infected children had milder symptoms and a better prognosis, and hence most of them improved rapidly [14]. Even though there is no evidence so far regarding the transplacental transmission of infection, more than 230 neonates have been infected [19]. Children are considered less vulnerable by some. Supporters of this idea attribute this to the assumption that children have limited interaction with the outer world compared to adults [27]. Hence, they are at less risk of developing the infection with no fatal outcomes [28]. However, some studies show that despite the limited exposure to the outside world, children are still at risk of getting the infection from their family members who are exposed to the environment.

Laboratory/Radiological Findings

The children who were hospitalized due to COVID-19 have shown various changes in their blood work. In a case series of nine pediatric patients, three patients showed leukopenia and lymphopenia, two showed increased lactate dehydrogenase (LDH), and nine had high C- reactive protein (CRP) and erythrocyte sedimentation rate (ESR) [25]. Another case series of children with COVID-19 showed normal blood work but changes on chest radiograph, which showed patchy infiltration [29]. One of the studies showed that pediatric patients with severe illness showed progressive lymphocytopenia with multiple changes on radiography. They showed pulmonary consolidations, bilateral ground-glass opacity, and infiltrating shadows with pleural effusion. Findings were more obvious on the CT scan, which showed consolidations bilaterally, and especially more towards the periphery [18].

In a study involving five children with COVID-19 with age ranging from ten months to six years, three children showed ground-glass opacities on CT, which were resolved with treatment [12]. A case report of a six-month-old boy in Singapore, who tested positive by real-time reverse transcription-polymerase chain reaction (RRT-PCR) with high viral load, showed neutropenia on day eight and even stool sample positive for COVID-19 on day nine in the hospital [30]. The CT scan of a 10-year old girl in Korea who presented with mild symptoms showed nodular consolidations with peripheral ground-glass opacities in the subpleural areas of the lower lobe on the right side [26]. Few patients also showed increased procalcitonin levels and consolidations with halo sign [31]. A study done in six provinces of China showed increased levels of procalcitonin, serum transaminase, and muscle enzymes in addition to increased ESR and CRP in the infected children. Their CT chest findings were mainly in the middle and outer zone of the lungs near the pleural area [7].

Comparison Between COVID-19 in Children and Adults

Is COVID-19 lenient with any age group? The answer is no. So far, we have seen that it has affected all age groups. Although the data for children with COVID-19 is limited, studies have shown that the pediatric population has indeed suffered from the infection with varying degrees of severity. However, some studies suggest that the infection is milder in children as compared to adults because of the difference in the characteristics of viral receptors [27]. Another difference was shown in a study performed on 13 pediatric patients who contracted the virus from the family cluster; the study showed typical findings on chest CT, which were consolidations with halo sign and were different from the findings in adults [31].

Treatment Modalities

The treatment for COVID-19 is still unclear, and there are no specific vaccines or medicines. From various studies, we have come to know that antivirals, antibacterials, immunoglobulins, antimalarials, high oxygen, and mechanical ventilation are used alone or in combination on a case-by-case basis. A study on newborns stated that one should consider giving high-dose pulmonary surfactant, nitric oxide inhalation, and high-frequency oscillatory ventilation to the newborns with acute respiratory distress syndrome [14]. In a case series of nine Iranian children with COVID-19, an improvement was achieved with supportive care without the need for ribavirin and mechanical ventilation [25]. Some critically ill pediatric patients were given antivirals [Virazole® (Bausch Health, Laval, Canada), oseltamivir, and interferon] and mechanical ventilation [22]. The antimalarial drug chloroquine phosphate has shown some efficacy and safety in COVID-19 patients during the clinical trials, which were conducted in China. And it is expected to be included in the guidelines for treating COVID-19 issued by the National Health Commission of the People’s Republic of China [32].

Besides the above treatments, self-isolation should be strictly maintained for COVID-19 patients. Symptomatic treatment still remains the mainstay of COVID-19 management.

Limitations

Although most of the data we collected was of high quality and appropriate, we still faced some limitations. Our main focus was on children with COVID-19, and limited data were available to assess the severity of the infection in that patient population. Moreover, most of the studies have been conducted in China, and not all of them are translated into English, which created some hindrance to our efforts to study the pediatric population.

## Conclusions

Our aim in writing this review article was to see whether or not COVID-19 has an impact on the pediatric population. Based on our findings, we have concluded that children are affected by the coronavirus in the same way as any other age group. Despite the limited data due to day-to-day changes in the statistics of patients infected, we have found that children, even if not infected themselves, can serve as carriers of the virus and can play a pivotal role in spreading the infection. The severity of symptoms in infected children varied from case to case. Therefore, further studies are required to gather enough data on children getting infected and also regarding the efficacy of different treatment modalities. We also learned that children usually have less exposure to this novel virus due to limited exposure to the external environment. However, they could still easily contract the disease from family members who traveled or got infected by other means. Therefore, our main purpose is to create awareness and lay emphasis on the importance of preventive measures that can be taken to avoid the spread of the infection.
